# Editorial: Nano-Biomaterials for the Delivery of Therapeutic and Biological Cues for Regenerative Medicine

**DOI:** 10.3389/fbioe.2021.832487

**Published:** 2022-01-13

**Authors:** Silvia Minardi

**Affiliations:** ^1^ Department of Orthopaedic Surgery, Northwestern University Feinberg School of Medicine, Chicago, IL, United States; ^2^ Simpson Querrey Institute of BioNanotechnology, Northwestern University, Chicago, IL, United States

**Keywords:** regenerative medicine, nanotechnology, biomaterials, drug delivery, progenitor cells

**Editorial on the Research Topic**

**Nano-Biomaterials for the Delivery of Therapeutic and Biological Cues for Regenerative Medicine**

Tissue damage can be the result of injury, disease or aging. Regenerative medicine aims at developing technologies to restore, maintain or augment tissue damage (Dimmeler et al., 2014). The ideal biomaterials for tissue regeneration should be both biocompatible and bioactive and able to harness the self-healing capabilities of the target tissue by providing crucial structural, compositional, and biochemical cues for repair.

Recently, the advantages of applying nanotechnology to regenerative medicine has become obvious, especially when examining nature. In fact, cells recognize chemical, physical, topographical and biological cues at the nanoscale, and nanotechnology allowed to engineer and fine tune biomaterials to mimic the nano-architecture and composition of target tissue (Liu et al., 2021). Consequently, this type of biomaterials has demonstrated superior ability to interface the host cells compared to conventional materials, resulting in overall improved regenerative outcomes (Abdollahiyan et al., 2021; Mohammadi et al., 2018). Numerous nano-structured biomaterials have been proposed as implantable devices for a variety of tissue engineering applications. For instance, bone is a natural nano-structured composite material (Minardi et al., 2015). 3D nanostructured biomaterials mimicking bone nano-structure and composition have been developed and tested in a variety of settings with success. Avitabile et al. showed how a biomimetic nanostructured composite was able to achieve enhanced osteogenic differentiation of progenitor cells, even in extreme physiological conditions such as microgravity. Additionally, the composite was also able to co-deliver not only bone-like chemical—physical cues, but also regenerative biological cues, resulting in enhanced bone augmentation in a posterolateral lumbar spinal fusion model, as described by Van Eps et al. In the review article by Lyons et al., nano-biomaterials for bone regeneration were comprehensively reviewed.

Nano-biomaterials have also been extensively explored as drug delivery carriers for their superior tunability. In fact, they can not only be fabricated in a variety of chemical compositions but also of shapes and sizes, as described by Melchor-Martínez et al. in their review article. An example of this regenerative strategy for bone is reported in the research article by Montagna et al., where the authors developed and tested novel strontium nanoparticles for the delivery of osteoinductive cues; nano-biomaterials for the delivery of therapeutics in the context of cardiovascular regeneration were reviewed by Zhu etal.

While initially nano-biomaterials were designed to interact mostly with progenitor cells, more recently, they have been engineered to interact specifically with the host immune cells, which was proven to enhance functional tissue regeneration (Rowley et al., 2019; Whitaker et al., 2021). Among these strategies, Sushnitha et al.review biomimetic nanoliposomes, with a focus on the potential of cell membrane-based nano-carriers, for their ability to favorably interact with the host (both systemically and locally) and even recognize and selectively target damaged tissue. Aguilar-Pérez et al. comprehensively reviewed the overall state-of-the-art of nanoliposomes as drug delivery carriers for regenerative medicine.

Despite all these advancements, a remaining challenge common to most fields of regenerative medicine is bacterial infections, which are frequent in cases of trauma or burns and can impair tissue regeneration (Bigham-Sadegh and Oryan, 2015; Naskar and Pharmaceutics, 2020). The clinical challenges posed by infections and the promise held by nano-biomaterials to address them were reviewed and discussed by Anastasio et al. The authors particularly highlighted nanoplatforms for the delivery of nitric oxide as both an antibacterial and pro-regenerative stimulus. An example of another nano-biomaterial targeting bacterial infections is reported in the research article by El-Deeb et al. The authors developed biologically synthesized silver nanoparticles able to reduce the number of infiltrating pro-inflammatory cells in a wound infection model. In summary, this research topic includes a collection of six review and four original research articles covering recent progress in nano-biomaterials design and their applications, particularly nanostructured materials able to deliver chemical, physical and biological cues to enhance tissue regeneration in a multiplicity of settings (e.g., bone, heart, wound healing).
